# Short-term metabolic changes during app-based dietary management in type 2 diabetes mellitus: a single-arm observational study with a separate Mendelian randomization analysis

**DOI:** 10.3389/fpubh.2026.1863236

**Published:** 2026-09-03

**Authors:** Dongping Xu, Yumei Qi, Ping Zheng, Huijuan Xiao, Linlin Shi, Xiaoxiao Tian, Yue Li, Xiaoli Zhou

**Affiliations:** 1 Department of Nutrition, Central Hospital, Tianjin University/Tianjin Third Central Hospital, Tianjin, China; 2 Tianjin Key Laboratory of Extracorporeal Life Support for Critical Diseases, Tianjin, China; 3 Tianjin Artificial Cell Engineering Technology Research Center, Tianjin, China; 4 Tianjin Institute of Geriatrics, Tianjin Third Central Hospital Branch, Tianjin, China; 5 Department of Toxicology, Tianjin Centers for Disease Control and Prevention, Tianjin, China

**Keywords:** digital nutrition management, glycemic control, Mendelian randomization, type 2 diabetes mellitus, visceral adiposity

## Abstract

**Background:**

We evaluated short-term metabolic changes during a structured app-based dietary management program in patients with type 2 diabetes mellitus (T2DM). In a separate analysis using independent genome-wide association study (GWAS) datasets, we examined the population-level association between genetically predicted liver fat and glycated hemoglobin (HbA1c).

**Methods:**

The clinical component was a prospective single-arm observational study of 103 adults with T2DM who received an individualized 4-week dietary prescription, recorded daily intake using a mobile application, and received weekly dietitian feedback. Anthropometric and laboratory measures were compared before and after the management period using paired tests. Associations between changes in glycemic and anthropometric indicators were assessed using Spearman correlation. The separate two-sample Mendelian randomization (MR) analysis used liver fat as the exposure and HbA1c as the outcome; inverse-variance weighted (IVW) analysis was primary.

**Results:**

Fasting blood glucose decreased from 7.26 ± 2.26 to 6.91 ± 2.08 mmol/L (*p* = 0.011), and HbA1c decreased from 6.86 ± 1.33% to 6.65 ± 1.10% (*p* < 0.001). Body weight and visceral fat area did not change significantly. The change in HbA1c was modestly correlated with the change in visceral fat area (*r* = 0.284, *p* = 0.016). In the separate MR analysis, the primary IVW estimate did not support an association between genetically predicted liver fat and HbA1c (*β* = 0.011, 95% CI − 0.019 to 0.041, *p* = 0.481). Nominal associations from secondary estimators were considered exploratory.

**Conclusion:**

Glycemic measures were lower after the 4-week management period than at baseline, but the single-arm design prevents attribution of these changes specifically to the program. The visceral fat–HbA1c correlation was exploratory. The separate MR analysis addressed a distinct population-level question and did not provide confirmatory evidence in the primary analysis.

## 1 Introduction

Type 2 diabetes mellitus (T2DM) is a highly prevalent chronic metabolic disorder. An estimated 537 million adults were living with diabetes worldwide in 2021, and this number is projected to reach 783 million by 2045 (1, 2). Medical nutrition therapy is a central component of T2DM management and can improve glycemic control when individualized and sustained (3, 4).

Traditional clinic-based nutrition care is limited by intermittent contact, recall bias in dietary assessment, and variable adherence (5). Mobile health applications can support real-time dietary logging, personalized feedback, and remote professional supervision (6). Limited peripheral adipose storage capacity has also been implicated in insulin resistance and ectopic fat accumulation (16). Systematic reviews suggest that smartphone-based self-management interventions can produce modest improvements in HbA1c, although effects vary across populations and programs (7, 8).

Visceral adipose tissue is metabolically active and may influence hepatic glucose metabolism through portal delivery of fatty acids and adipokines (9). However, an association between changes in visceral adiposity and glycemic measures during a short management period does not establish mediation or causality, particularly in an uncontrolled pre-post study.

Mendelian randomization (MR) uses genetic variants as instrumental variables to evaluate exposure-outcome associations under specific assumptions (10). In the present work, the clinical cohort and GWAS samples were independent, and the adiposity phenotypes differed: visceral fat area was estimated by bioelectrical impedance in the clinical cohort, whereas liver fat was derived from magnetic resonance imaging in the GWAS. The MR analysis was therefore not designed to explain, validate, or refute the clinical findings; it addressed a separate population-level question.

The study had two independent aims: first, to describe short-term metabolic changes during a 4-week app-based dietary management period in patients with T2DM and explore correlations between changes in glycemic and anthropometric measures; and second, to examine the association between genetically predicted liver fat and HbA1c using two-sample MR.

## 2 Methods

### 2.1 Study design and participants

The study comprised two independent components: a prospective single-arm observational clinical study and a separate two-sample MR analysis. For the clinical component, 103 adults with T2DM were enrolled from six urban districts of Tianjin, China, between November 2019 and September 2022. Inclusion criteria were age 18–65 years, a confirmed diagnosis of T2DM according to World Health Organization criteria, body mass index of 18.5–27.9 kg/m^2^, possession of a compatible smartphone, and written informed consent. Exclusion criteria included type 1 diabetes, gestational diabetes, severe hepatic or renal dysfunction, active malignancy, pregnancy or lactation, and inability to complete the dietary recording protocol.

### 2.2 App-based dietary management

**Figure 1:**
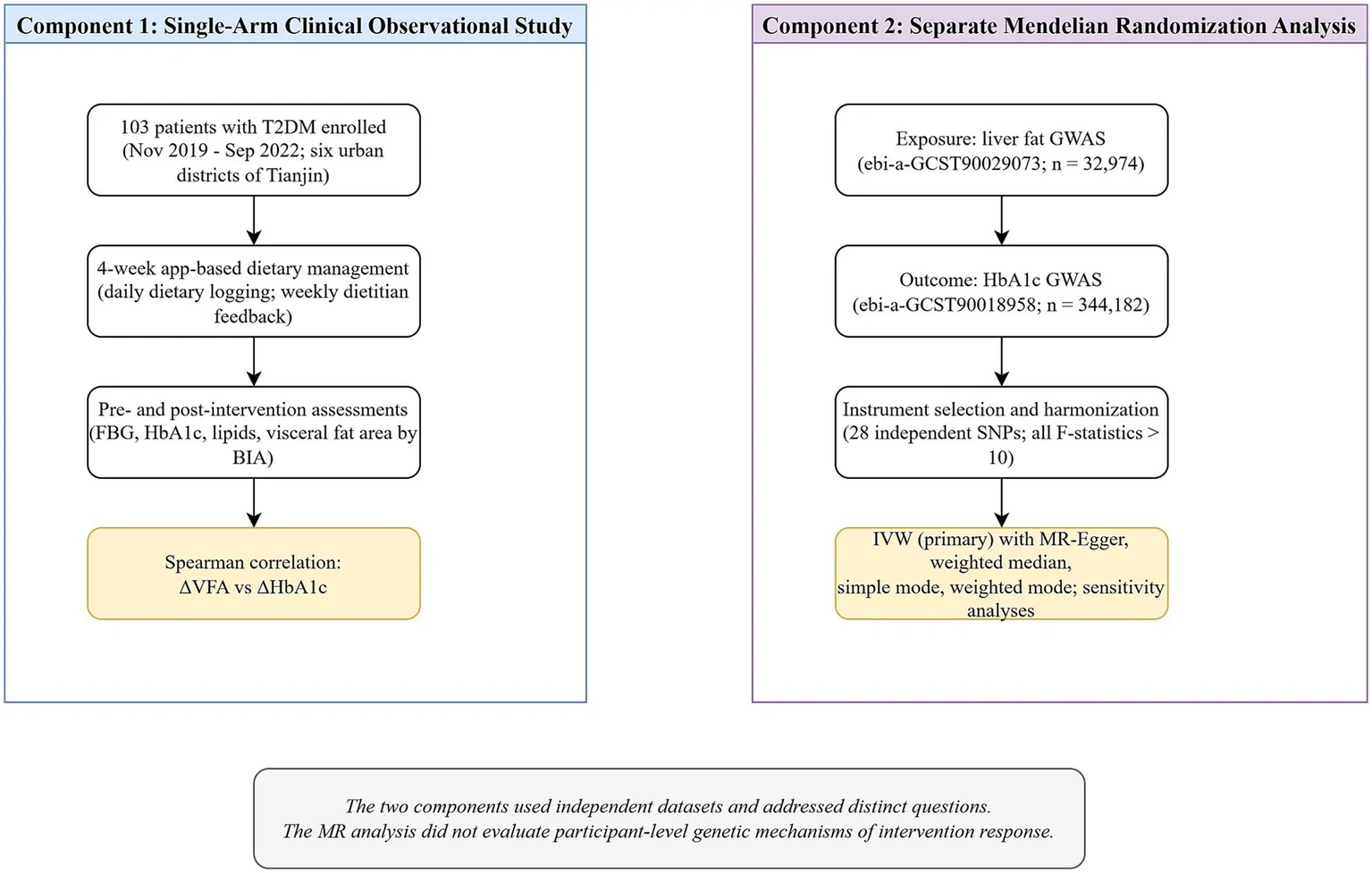
Study overview. The clinical component was a prospective single-arm observational study of 103 patients with T2DM undergoing 4 weeks of app-based dietary management. The separate MR component used independent GWAS summary statistics for liver fat (ebi-a-GCST90029073) and HbA1c (ebi-a-GCST90018958). The two components used independent datasets, examined different adiposity phenotypes, and did not evaluate participant-level genetic mechanisms of response.

Participants received individualized 4-week dietary prescriptions developed by registered dietitians in accordance with Chinese dietary guidance for T2DM. Daily dietary intake was recorded using the Health Link mobile platform, which supported macronutrient analysis and portion-size estimation. Dietitians reviewed the records and provided weekly feedback and dietary adjustments through the application Figure 1.

### 2.3 Clinical measurements

Anthropometric and laboratory assessments were performed at baseline and after 4 weeks. Body weight, body fat percentage, and visceral fat area were assessed using bioelectrical impedance analysis (InBody 770, Biospace, Seoul, Korea). Waist-to-hip ratio was calculated from standardized measurements. After an overnight fast of at least 8 h, fasting blood glucose (FBG), total cholesterol, triglycerides, high-density lipoprotein cholesterol, low-density lipoprotein cholesterol, total protein, and albumin were measured using an automated biochemical analyzer (Roche Cobas 8,000, Roche Diagnostics, Basel, Switzerland). HbA1c was measured by high-performance liquid chromatography (Bio-Rad Variant II, Bio-Rad Laboratories, Hercules, CA, United States).

### 2.4 Statistical analysis of the clinical component

This was an exploratory study; no formal *a priori* sample size calculation was performed, and the sample size was determined by enrollment during the study period. Continuous variables are presented as mean ± standard deviation. Pre and post-management measurements were compared using two-tailed paired Student’s *t*-tests. Associations between changes (post-management minus baseline, denoted Δ) were assessed using Spearman rank correlation coefficients. Analyses used available paired observations for each measure. Because multiple exploratory correlations were examined, no formal multiplicity adjustment was applied; the results should therefore be interpreted cautiously. A two-tailed *p* < 0.05 was considered statistically significant. Clinical analyses were performed using SPSS version 26.0 (IBM Corp., Armonk, NY, United States).

### 2.5 Mendelian randomization analysis

The separate two-sample MR analysis examined the association between genetically predicted liver fat and HbA1c. Summary statistics for MRI-derived liver fat were obtained from 32,974 participants of European ancestry (GWAS ID: ebi-a-GCST90029073) (11). Summary statistics for HbA1c were obtained from 344,182 participants of European ancestry (GWAS ID: ebi-a-GCST90018958) (12). At the conventional threshold of *p* < 5 × 10^−8^, too few independent variants remained after linkage disequilibrium clumping for the planned sensitivity analyses. An exploratory instrument set was therefore constructed using *p* < 5 × 10^−6^. After clumping (*r*^2^ < 0.001 within 10,000 kb) and harmonization, 28 independent variants were retained. F-statistics ranged from 21 to 87 (mean 34), exceeding the conventional threshold of 10.

The IVW method under a random-effects model was the primary estimator. MR-Egger regression, weighted median, simple mode, and weighted mode were used as secondary estimators. Heterogeneity was assessed using Cochran’s *Q* test, directional horizontal pleiotropy using the MR-Egger intercept, and influence of individual variants using leave-one-out analysis. Analyses were performed using the TwoSampleMR package (version 0.5.6) in R (version 4.2.1). Because HbA1c was analyzed as a continuous trait, estimates are reported as *β* coefficients with 95% confidence intervals (CIs) and *p* values on the scale of the outcome GWAS.

### 2.6 Ethics statement

The clinical protocol was approved by the Institutional Review Board and Ethics Committee of Tianjin Third Central Hospital (Approval Number: SZX-IRB-SOP-016(F)-002-02) and was conducted in accordance with the Declaration of Helsinki. All participants provided written informed consent. The MR component used publicly available de-identified summary statistics and required no additional ethical approval.

## 3 Results

### 3.1 Study population and data availability

A total of 103 participants were enrolled. The number of paired observations varied across measures because of incomplete follow-up measurements: 93 participants had paired anthropometric measurements, 96 had paired biochemical measurements including FBG, 76 had paired HbA1c measurements, and 72 had paired visceral fat area measurements (Tables 1, 2).

**Table 1:** Anthropometric, nutritional, and lipid indicators before and after the 4-week management period.

Indicator	*N*	Baseline, mean ± SD	Week 4, mean ± SD	*p* value
Body weight (kg)	93	68.08 ± 12.68	66.77 ± 13.75	0.120
Body fat percentage (%)	93	31.30 ± 7.46	30.49 ± 11.98	0.438
Waist-to-hip ratio	93	1.29 ± 3.74	1.30 ± 3.84	0.476
Visceral fat area (cm^2^)	72	109.58 ± 32.93	109.28 ± 31.89	0.772
Total protein (g/L)	96	72.35 ± 4.26	72.14 ± 3.71	0.542
Albumin (g/L)	96	45.27 ± 2.88	50.06 ± 43.89	0.287
Triglycerides (mmol/L)	96	1.74 ± 1.03	1.62 ± 0.71	0.204
Total cholesterol (mmol/L)	96	4.88 ± 1.00	4.80 ± 0.99	0.291
HDL cholesterol (mmol/L)	96	1.16 ± 0.25	1.16 ± 0.28	0.981
LDL cholesterol (mmol/L)	96	2.88 ± 0.80	2.87 ± 0.92	0.863

*p* values were calculated using two-tailed paired Student’s *t*-tests. HDL, high-density lipoprotein; LDL, low-density lipoprotein; SD, standard deviation.

**Table 2:** Glycemic indicators before and after the 4-week management period.

Indicator	*N*	Baseline, mean ± SD	Week 4, mean ± SD	Mean difference	*p* value
Fasting blood glucose (mmol/L)	96	7.26 ± 2.26	6.91 ± 2.08	−0.35	0.011
HbA1c (%)	76	6.86 ± 1.33	6.65 ± 1.10	−0.21	<0.001

*p* values were calculated using two-tailed paired Student’s *t*-tests. HbA1c, glycated hemoglobin; SD, standard deviation.

### 3.2 Anthropometric, nutritional, and lipid indicators

No statistically significant group-level differences were observed in body weight, body fat percentage, waist-to-hip ratio, visceral fat area, total protein, albumin, triglycerides, total cholesterol, high-density lipoprotein cholesterol, or low-density lipoprotein cholesterol after the 4-week period (all *p* > 0.05; Table 1).

### 3.3 Glycemic indicators

**Figure 2:**
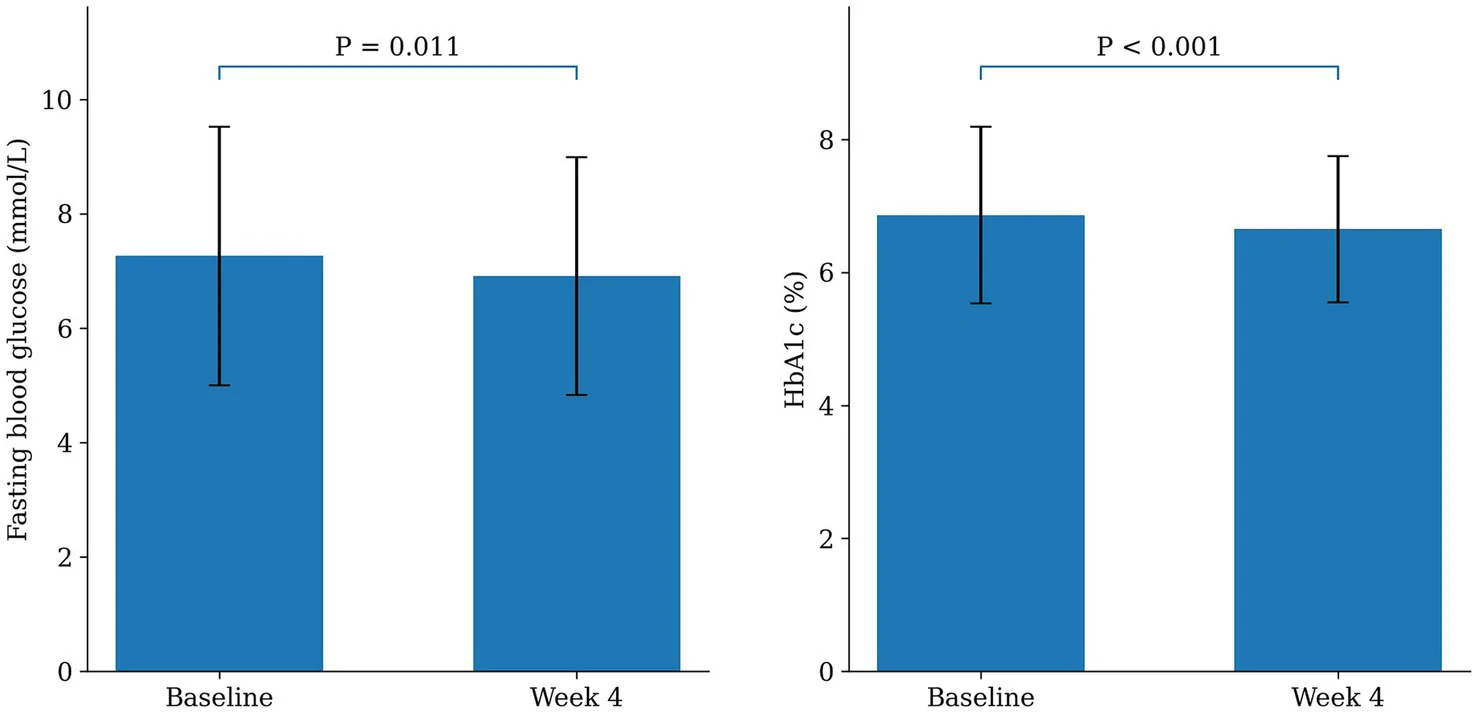
Fasting blood glucose and HbA1c at baseline and after the 4-week app-based dietary management period. Data are presented as mean ± standard deviation. Paired comparisons yielded *p* = 0.011 for fasting blood glucose and *p* < 0.001 for HbA1c.

FBG was 6.91 ± 2.08 mmol/L after 4 weeks compared with 7.26 ± 2.26 mmol/L at baseline (mean difference −0.35 mmol/L, *p* = 0.011). HbA1c was 6.65 ± 1.10% compared with 6.86 ± 1.33% at baseline (mean difference −0.21 percentage points, *p* < 0.001) (Table 2; Figure 2).

### 3.4 Exploratory correlation analyses

Changes in FBG were not significantly correlated with changes in body weight, lipid measures, or body fat percentage (all *p* > 0.05; Table 3). The change in HbA1c was not significantly correlated with changes in body weight, albumin, or total protein. A modest positive correlation was observed between ΔHbA1c and Δvisceral fat area (Spearman *r* = 0.284, *p* = 0.016; Table 4). This unadjusted association was considered exploratory.

**Table 3:** Exploratory spearman correlations between ΔFBG and changes in metabolic indicators.

Indicator	Spearman *r*	*p* value
ΔBody weight	0.011	0.915
ΔTriglycerides	0.036	0.729
ΔTotal cholesterol	0.121	0.240
ΔHDL cholesterol	0.091	0.376
ΔLDL cholesterol	0.034	0.740
ΔBody fat percentage	0.025	0.809

Δ denotes week 4 minus baseline. FBG, fasting blood glucose; HDL, high-density lipoprotein; LDL, low-density lipoprotein.

**Table 4:** Exploratory spearman correlations between ΔHbA1c and changes in metabolic indicators.

Indicator	Spearman *r*	*P* value
ΔBody weight	−0.099	0.344
ΔAlbumin	−0.064	0.538
ΔTotal protein	0.003	0.980
ΔVisceral fat area	0.284	0.016

Δ denotes week 4 minus baseline. HbA1c, glycated hemoglobin. Correlation analyses were exploratory and were not adjusted for multiple comparisons.

### 3.5 Mendelian randomization analysis

**Figure 3:**
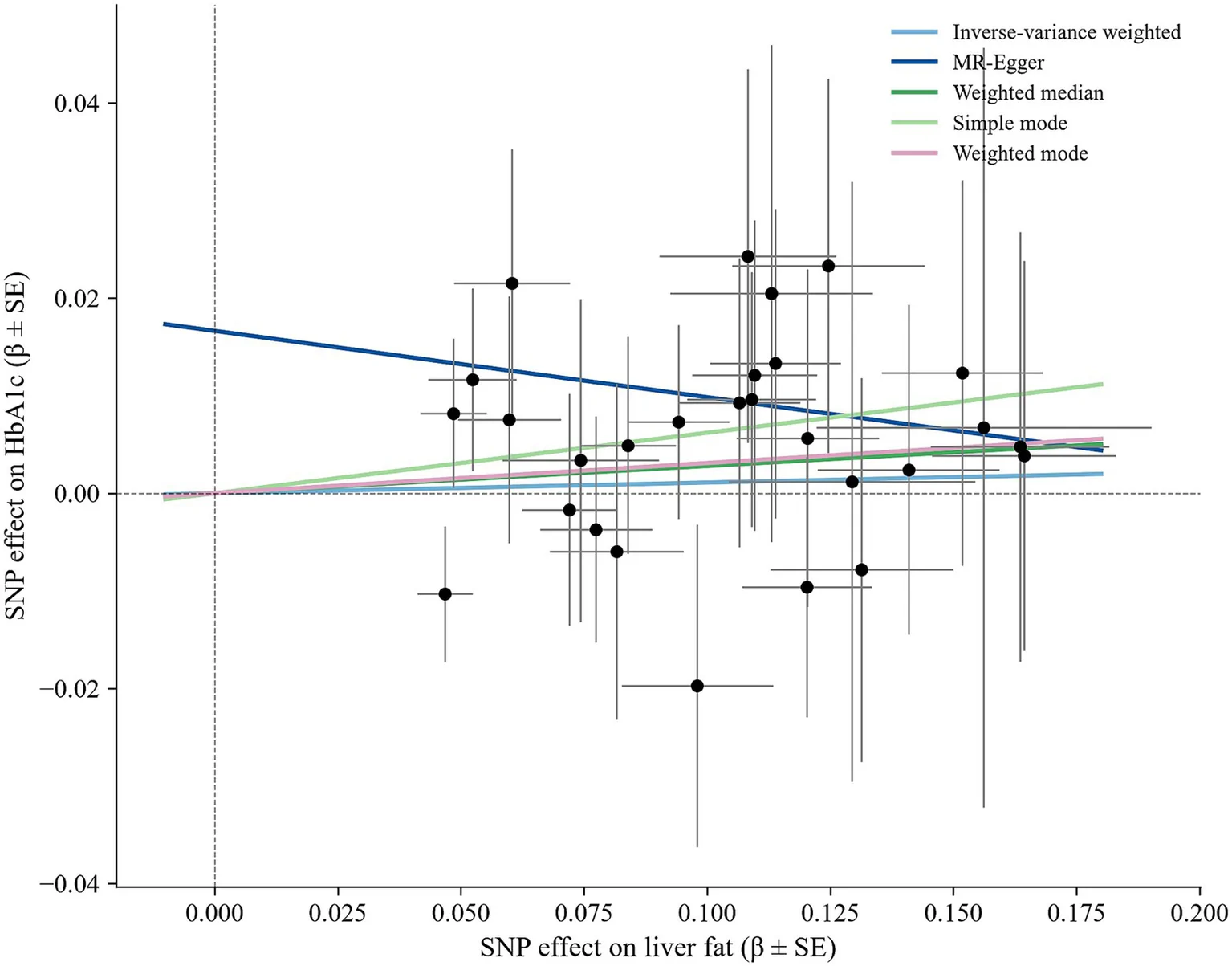
Scatter plot of SNP effects on liver fat and HbA1c. Points represent individual genetic instruments with standard errors; lines show estimates from the inverse-variance weighted, MR-Egger, weighted median, simple mode, and weighted mode methods.

**Figure 4:**
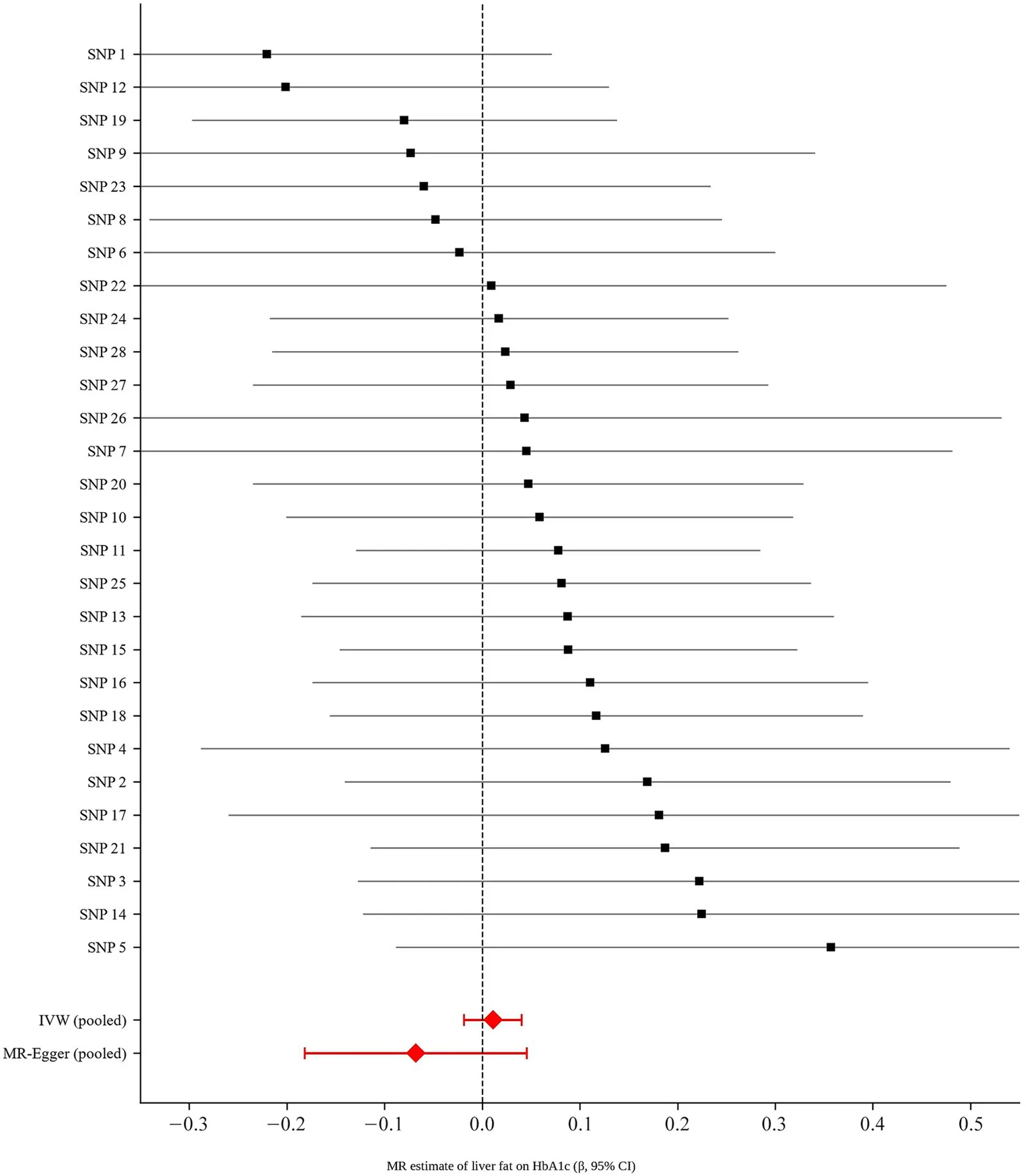
Forest plot of individual-variant and pooled MR estimates for liver fat and HbA1c. Points and horizontal lines represent *β* estimates and 95% confidence intervals. Pooled inverse-variance weighted and MR-Egger estimates are shown at the bottom.

**Figure 5:**
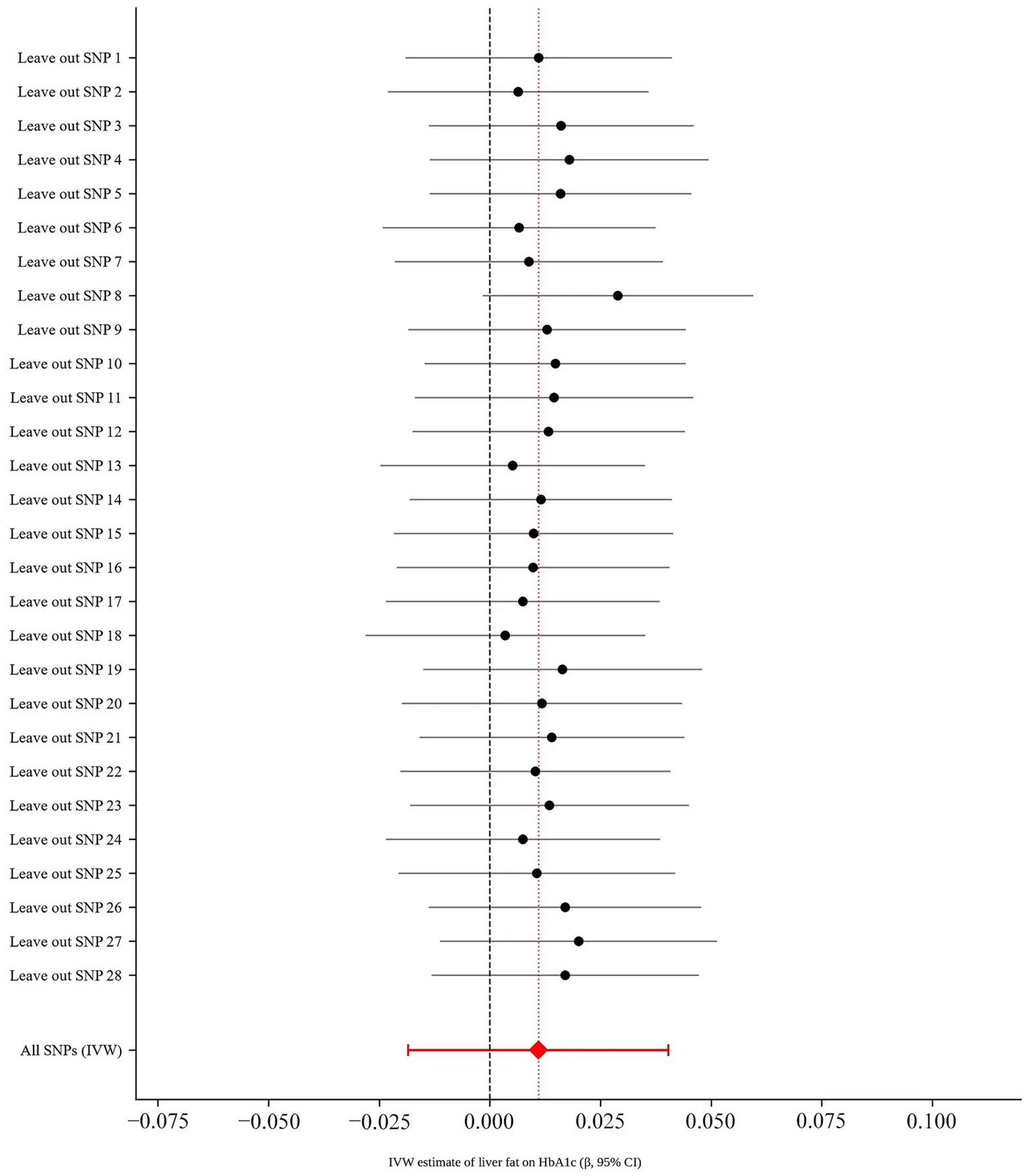
Leave-one-out analysis of the inverse-variance weighted estimate. Each row shows the pooled *β* estimate after exclusion of one instrument; the bottom row shows the estimate using all instruments.

The primary IVW estimate did not support an association between genetically predicted liver fat and HbA1c (*β* = 0.011, 95% CI − 0.019 to 0.041, *p* = 0.481). The MR-Egger estimate was also non-significant (*β* = −0.068, 95% CI − 0.190 to 0.054, *p* = 0.276). The weighted median (*β* = 0.028, 95% CI 0.002 to 0.054, *p* = 0.034) and simple mode (*β* = 0.062, 95% CI 0.008 to 0.116, *p* = 0.025) produced nominal associations. Because IVW was the pre-specified primary estimator, secondary estimates were considered hypothesis-generating rather than confirmatory (Table 5; Figures 3, 4). Cochran’s *Q* test indicated heterogeneity (*p* = 0.013), whereas the MR-Egger intercept did not indicate directional horizontal pleiotropy (*p* = 0.112). Leave-one-out analysis did not identify a single variant that materially changed the overall IVW estimate (Figure 5).

**Table 5:** Mendelian randomization estimates for genetically predicted liver fat and HbA1c.

Estimator	*β*	95% CI	*P* value	Interpretation
Inverse-variance weighted	0.011	−0.019 to 0.041	0.481	Primary; null
MR-Egger	−0.068	−0.190 to 0.054	0.276	Secondary; null
Weighted median	0.028	0.002 to 0.054	0.034	Secondary; exploratory
Simple mode	0.062	0.008 to 0.116	0.025	Secondary; exploratory

Estimates are reported on the *β* scale of the continuous HbA1c GWAS. CI, confidence interval; HbA1c, glycated hemoglobin; MR, Mendelian randomization.

## 4 Discussion

In the clinical component, FBG and HbA1c were lower after the 4-week app-based dietary management period than at baseline. Body weight and visceral fat area did not show significant group-level changes. At the individual level, ΔHbA1c was modestly correlated with Δvisceral fat area, but this unadjusted finding does not establish that visceral fat change mediated the glycemic change.

A difference in HbA1c over 4 weeks requires cautious interpretation. HbA1c generally reflects glycemic exposure over approximately 8–12 weeks, although more recent glucose levels contribute disproportionately to the measured value (13, 14). The observed difference may therefore reflect recent glycemic exposure, but postprandial glucose excursions, continuous glucose monitoring metrics, and fructosamine were not measured. The study duration was also insufficient to assess persistence of the change.

The observed correlation between ΔHbA1c and Δvisceral fat area was statistically significant but modest. The portal hypothesis provides a biologically plausible framework because visceral adipose tissue has direct portal venous drainage and may influence hepatic metabolism (9). However, the study did not measure portal free fatty acid flux, hepatic glucose production, or insulin sensitivity. Moreover, the absence of a statistically significant weight correlation does not demonstrate that body weight was unimportant; the range of change, measurement error, residual confounding, and limited statistical power may have influenced the findings.

The separate MR analysis addressed whether genetically predicted liver fat was associated with HbA1c in independent European-ancestry GWAS datasets. The primary IVW estimate was null, whereas nominal associations from two secondary estimators were exploratory. Because the MR exposure differed from the visceral fat phenotype measured in the clinical cohort and the two components used unrelated datasets, the MR findings should not be interpreted as confirming or refuting the clinical correlation. The relaxed instrument threshold, heterogeneity, and ancestry restriction further limit the precision and generalizability of the MR results.

Several limitations should be acknowledged. First, the single-arm pre-post design lacked a control group, preventing attribution of the observed glycemic differences specifically to the app-based program. Regression to the mean, the Hawthorne effect, concurrent treatment adherence, spontaneous changes in diet or physical activity, and other unmeasured factors remain alternative explanations (15). Second, detailed diabetes duration, antidiabetic medication classes, insulin and GLP-1 receptor agonist use, and quantitative adherence were not consistently available in the analyzable dataset. Multivariable adjustment was therefore not performed, and residual confounding is likely. Third, no formal *a priori* sample size calculation or multiplicity correction was applied. Fourth, visceral fat area was estimated by bioelectrical impedance rather than computed tomography or magnetic resonance imaging. Fifth, the observation period was short. Finally, the MR component used European-ancestry GWAS data and an exploratory instrument threshold.

The study nevertheless provides a real-world description of short-term metabolic measurements during app-supported dietary management and explicitly separates the clinical observation from the independent population-genetic analysis. The results support further evaluation in controlled studies with pre-specified sample-size calculations, standardized adherence measures, detailed medication data, and longer follow-up.

## 5 Conclusion

In this single-arm observational study, FBG and HbA1c were lower after 4 weeks of app-based dietary management than at baseline, but the design does not permit attribution of these differences specifically to the program. The modest unadjusted association between changes in visceral fat area and HbA1c was exploratory. In the separate MR analysis, the primary IVW estimate did not support an association between genetically predicted liver fat and HbA1c. The clinical and MR components addressed distinct questions and should be interpreted independently. Randomized controlled studies with longer follow-up and more complete covariate data are required.

## Data Availability

The original contributions presented in the study are included in the article/supplementary material, further inquiries can be directed to the corresponding author.
